# Supplementary material for: The genome sequence of the Common Flat-body moth, Agonopterix heracliana Linnaeus, 1758
Source: Wellcome Open Res. 2024 Jun 21;9:329. [Version 1] doi: 10.12688/wellcomeopenres.22461.1 (PMC11470982; doi:10.12688/wellcomeopenres.22461.1)
Supplement: Peer review history

# Reviewer report: Reviewer response for version 1

**DOI:** 10.21956/wellcomeopenres.24748.r101601

**Published:** 2024-10-12

**Authors:** Marta Vila

**Affiliations:** 1 Universidade da Coruna, A Coruña, Galicia, Spain

*Agonopterix heracliana* (Linnaeus, 1758) is a common species of moth in the family Depressariidae, widely distributed across the UK and much of Europe. Commonly referred to as the "Common Flat-body" or "Common Brindled Brown", this species is often associated with umbelliferous plants, such as common hogweed *Heracleum sphondylium* (hence, the "heracliana") and cow parsley ( *Anthriscus sylvestris*), where the larvae feed on leaves. Although *A. heracliana* does not pose significant agricultural threats, it plays an important ecological role as a herbivore in various ecosystems. Given its wide distribution and interactions with various plant species, this genome assembly represents a valuable resource for future ecological, evolutionary, and comparative genomic studies.

 The sequencing and assembly of the genome of *A. heracliana* provide a foundational resource for investigating a range of biological questions. As one of the first genomic resources for a species within the Depressariidae family, this genome will allow researchers to explore the evolutionary relationships between this species and other Lepidoptera, particularly in understanding divergence patterns within the superfamily Gelechioidea. Furthermore, the availability of this genome will be crucial for examining the molecular basis of ecological adaptations, such as host plant selection and tolerance to environmental stressors.

 The manuscript follows the typical structure for a Genome Note, and the procedures are appropriate. My comments are as follows:

1. **Background Section:** I would suggest a minor change in this sentence “… *heracliana* can be difficult to distinguish **in the UK **from *A. ciliella*”. This is because a third, very similar species, *A. caucasiella*, is also present, though it is primarily distributed in the Caucasus region (Karsholt et al. 2006).
2. **Genome Sequence Report Section:** The authors should acknowledge that sequencing a female specimen is necessary to provide information about the W chromosome.|
3. **Genome Sequence Report Section:** I would appreciate a brief explanation of how future research could address the issue of the Z chromosome not being fully phased. The sentence "While not fully phased, the assembly deposited is of one haplotype. Contigs corresponding to the second haplotype have also been deposited" could be expanded to clarify how this challenge might be resolved in the future.

Are sufficient details of methods and materials provided to allow replication by others?

Yes

Is the rationale for creating the dataset(s) clearly described?

Yes

Are the datasets clearly presented in a useable and accessible format?

Yes

Are the protocols appropriate and is the work technically sound?

Yes

Reviewer Expertise:

Molecular Ecology, Phylogeography

I confirm that I have read this submission and believe that I have an appropriate level of expertise to confirm that it is of an acceptable scientific standard.

# Reviewer report: Reviewer response for version 1

**DOI:** 10.21956/wellcomeopenres.24748.r94991

**Published:** 2024-10-09

**Authors:** Daniel Doucet

**Affiliations:** 1 Canadian Forest Service, Natural Resources Canada, Ontario, Canada

The report by Boyes and colleagues describes the complete genome sequencing of the moth *Agonopterix heracliana. *The Depressariidae is a moderately speciose family of moths (approximately 2,300) within the Gelechioidea superfamily. The Gelechioidea are known to display very diverse life history strategies, such as predation or living in aquatic environments. Understanding the adaptive radiation of this group benefits from the provision of complete genomes from as many species as possible, such as *A. heracliana.* 

 The rationale for creating the dataset for this species is well described. The host plants that this species feeds on (Apiacae) are know to synthesize a wide array of secondary metabolites, some of which function as defensive compounds. It will be interesting to examine the genome for genes encoding detoxification enzymes that may act against Apiacae defenses. 

 The collection, labeling and processing of the insect sample is appropriate and follows the standard of the field in genomics. Although not an expert in bioinformatics, the work follows the standard workflow established by the Tree of Life, with thousands of genomes assembled using the same methods. The methods are described in sufficient  details so that the work can be replicated. Likewise the datasets are clearly presented and accessible in a useable format.

Are sufficient details of methods and materials provided to allow replication by others?

Yes

Is the rationale for creating the dataset(s) clearly described?

Yes

Are the datasets clearly presented in a useable and accessible format?

Yes

Are the protocols appropriate and is the work technically sound?

Yes

Reviewer Expertise:

Cell biology

I confirm that I have read this submission and believe that I have an appropriate level of expertise to confirm that it is of an acceptable scientific standard.
